# Influence of hypoxia on biochemical aspects and on expression of genes related to oxygen-homeostasis of the Amazonian cichlid *Astronotus ocellatus* (Agassiz, 1831)

**DOI:** 10.1590/1678-4685-GMB-2021-0127

**Published:** 2021-11-19

**Authors:** José L. Vasconcelos-Lima, Vanessa L. Oikawa-Cardoso, Waldir Heinrichs-Caldas, Vera M. F. Almeida-Val

**Affiliations:** 1 Instituto Nacional de Pesquisas da Amazônia, Laboratório de Ecofisiologia e Evolução Molecular (LEEM), Manaus, AM, Brazil.

**Keywords:** Amazon, glucose, glycogen, hif-1α, phd2

## Abstract

Variations in dissolved oxygen levels are common in the Amazonian aquatic environments and the aquatic organisms that inhabit these environments developed a variety of adaptive responses to deal with such conditions. Some Amazonian fish species are tolerant to low oxygen levels and the cichlid *Astronotus ocellatus* is one of the most hypoxia-tolerant species. Herein, we aimed to unveil the biochemical and molecular responses that *A. ocellatus* presents when submitted to hypoxia. Hypoxia indicators were measured, such as plasma glucose, plasma lactate, hepatic glycogen and relative transcript levels of prolyl hydroxylase 2 (*phd2)* and hypoxia-inducible factor-1α (*hif-1α*) in juveniles of approximately 50 g exposed to 1, 3, and 5 hours of hypoxia (0.7 mg O_2_.L^-1^), followed by 3 hours of recovery in normoxia (6 mg O_2_.L^-1^). Fish exposed to hypoxia reduced liver glycogen levels within 3 hours of hypoxia, when comparing with 1 hour, and increased plasma glucose and lactate. Under the same condition, *phd2* transcripts levels increased in gills, but decreased in liver. In contrast, hypoxia did not affect relative gene expression of *hif-1α* in both tissues. Based on the transcription pattern of *phd2*, these results showed that liver and gills of *A. ocellatus* have different molecular strategies to cope with environmental hypoxia.

## Introduction

The annual and regular variation of water level of the Amazon basin rivers described by Junk et al. (1989) as the “flood pulse” is the main driving force responsible for the productivity, existence and interaction between the biota and the river-floodplain system. Selective pressures for adaptations can be explained by various environmental challenges faced by these organisms during their lifespan, like short- and long-term changes in water pH, ion availability, temperature and availability of dissolved oxygen (Almeida-Val et al., 1999). The Amazonian fishes present biochemical, physiological and behavioral adaptations to deal with hypoxic environments (Val *et al*., 1998). These adaptive responses can be related to reduced energy demand, improved oxygen uptake, or simply avoidance of hypoxic areas (Val, 1995; Muusze et al., 1998; Almeida-Val *et al*., 1999; Sloman et al., 2006). Although hypoxia is not lethal for some fish species, sublethal effects may influence the biological functions of an organism and, consequently, its fitness (Cheung et al., 2014). Compared to other Amazonian fishes, *Astronotus ocellatus* (also known as Oscar fish) is a highly hypoxia tolerant Cichlidae species (Muusze *et al*., 1998; Sloman *et al*., 2006). Adult individuals can tolerate 5-20% of air-saturated oxygen tension for a period of 20 to 50 hours and, in anoxic condition, their tolerance period is up to 6 hours at 28 °C (Muusze *et al*., 1998). This high hypoxia tolerance is product of a series of physiological adjustments aimed to reduce the oxygen demand. The remarkable hypoxia tolerance of *Astronotus ocellatus* is primarily based on the aerobic metabolism depression followed by an activation of the anaerobic glycolysis (Muusze *et al*., 1998; Scott et al., 2008). When exposed to hypoxia in the wild, *A. ocellatus* remains in place, an ecological behavior allowed by its adaptations and high tolerance to hypoxia.

Organisms under hypoxia show numerous physiological and molecular outcomes of the expression of several genes regulated by hypoxia with fundamental importance for its homeostasis (Nikinmaa and Rees, 2005). The hypoxia-inducible transcription factor (HIF) is stabilized in response to reductions of oxygen levels (Maxwell et al., 1999). HIF-1 is a heterodimer protein composed of two subunits, HIF1-α and HIF1-β, expressed by the *hif-1α* and *hif-1β* genes, respectively. Both subunits contain a basic helix-loop-helix (bHLH) and a PAS domain (Per, ARNT, Sim), required for heterodimerization, DNA binding and transactivation (Kewley et al., 2004; Nikinmaa and Rees, 2005). The protein HIF-1 binds to specific regions of the DNA and regulates the transcription of oxygen-regulated genes (Wenger et al., 2005).

In fish, some target genes are known, such as erythropoietin (*epo*), glucose transporter 1 (*slc2a1*), lactate dehydrogenase B (*ldhb*), and vascular endothelial growth factor A (*vegfa*) (Rashid et al., 2017). According to Kodama et al. (2012), HIF is an evolutionarily conserved in vertebrates, acting as the main regulator of gene expression in cells exposed to hypoxia.

Studies with vertebrates indicate that the HIF-1α subunit is constantly synthesized, being rapidly degraded under normal oxygen conditions (Semenza, 2004). Its degradation is mediated by a specific region called oxygen-dependent degradation domain (ODD). Under normal oxygen condition, this domain has two conserved proline residues, which are covalently modified by the action of the prolyl-hydroxylase (PHD) (Nikinmaa and Rees, 2005). There are three functional isoforms of the PHD enzyme: PHD1, PHD2, and PHD3, expressed by *phd1*, *phd2*, and *phd3* genes, respectively. Each isoform differs in the mRNA relative abundance, but all forms show the same pattern of ubiquitous expression in human cells (Cioffi et al., 2003). Among the three PHD isoforms in human cells, PHD2 has the highest affinity for HIF-1α (Berra et al., 2003; Appelhoff et al., 2004). PHDs detect and utilize oxygen as substrate to insert the hydroxyl group onto the proline residues of the HIF-1α subunit. The hydroxylation reaction of PHDs requires 2-oxoglutarate and iron as cofactors. When HIF proline residues are hydroxylated, HIF-1α is recognized by the von-Hippel-Lindau (pVHL) protein, ubiquitinated and degraded by the proteasome pathway in the 26S proteasome (Epstein et al., 2001; Nikinmaa and Rees, 2005; Kaelin Jr and Ratcliffe, 2008; Rytkönen et al., 2011).

In hypoxic conditions, PHD enzyme activity levels are inhibited due to the low oxygen saturation (Nikinmaa and Rees, 2005). Thus, HIF-1α protein is stabilized, accumulates and is transferred from the cytoplasm to the nucleus, where it binds to the HIF-1β subunit, forming HIF-1 transcription factor. HIF-1 will associate with the general transcription factors (CBP/p300) and bind to Hypoxia Responsive Elements (HRE), which are conserved sequences located in the promoter regions of induced genes by hypoxia. Thus, HIF-1 target genes are related to the processes of angiogenesis, erythropoiesis, glycolysis, iron transport, apoptosis and cell cycle control (Nikinmaa and Rees, 2005; Kaelin Jr and Ratcliffe, 2008; Rytkönen et al., 2011).

Considering the high hypoxia tolerance of Oscars in nature and laboratory experiments, and considering the above described processes of PHD-HIF oxygen-sensing system, since this is responsible for most of hypoxia responsive elements, in this study we aimed to understand how these two genes contribute to the adaptive responses to hypoxia in *Astronotus ocellatus* and how they might be involved in this animal’s hypoxia responses, such as the switch to anaerobic metabolism and metabolic depression.

## Material and Methods

### Experimental animals

Juveniles of *A. ocellatus* (5 g ± 0.5) were purchased from a commercial supplier (Fish Farm Santo Antônio, Rio Preto da Eva City, Amazonas, Brazil), and transferred to the Laboratory of Ecophysiology and Molecular Evolution (LEEM) at National Institute for Amazon Research (INPA), Manaus, Amazonas, Brazil, for acclimation. The animals were held outdoors in 500 L tanks with aerated water (approximately 7 mg O_2_.L^-1^) at 27º C ± 2, pH varying between 5 and 6, under constant water renewal and natural light exposure. The animals were reared in this condition during approximately three months until they reached the experimental weight, which was 50 grams. Fishes were fed once a day until satiation with commercial pelleted food (36% protein). Feeding was suspended 24 hours prior the experiments.

### Experimental design: Hypoxia exposure and recovery in normoxia

The experimental protocol was carried out in accordance with the Brazilian Guidelines for Use and Care of Animals (CONCEA), with the authorization of INPA’s Committee for Ethics in Animal Use (CEUA protocol #022/2017), and no fish died during the hypoxia exposition.

Forty-eight juveniles of *A. ocellatus*, weighing 49.6 ± 0.9 g, were used. For the experiment, two 100 L tanks were used and four fish were placed in each tank 24 hours prior the beginning of the experiment for acclimation. Water temperature was maintained at 28 ºC and the whole experiment was carried out indoors. The animals were kept separated by grids so they did not have contact with each other. For hypoxia treatment, the aeration of one tank was interrupted and N_2_ gas was pumped into the water until the oxygen level reached 0.7 mg O_2_.L^-1^ ± 0.5. The tank was covered with bubble wrap to prevent oxygen diffusion from the air. The animals were submitted to hypoxia for 1 hour, 3 hours and 5 hours, followed by 3 hours of recovery when the aeration was taken back to normoxic concentration (6 mg O_2_.L^-1^ ± 0.5). For the control, the aeration of the other tank were constant throughout the whole experiment (6 mg O_2_.L^-1^). One animal from each tank was sampled after 1, 3, and 5 hours, followed by 3 hours of recovery. This experiment was repeated six times, thus six animals was sampled for each period. At the end of the experiment, twenty-four animals were used for control and twenty-four animals for hypoxia treatment. 

In order to assure the animals were in hypoxia, the oxygen concentration for hypoxia treatment was approximately half of the critical oxygen level (*PO*_*2crit*_ ) value found by De Boeck et al. (2013) for *A. ocellatus* and was the concentration was similar to the one used by Baptista et al. (2016). Three hours of recovery in normoxia was chosen because this recovery period was enough for protein synthesis to return to normal levels in gills and liver of *A. ocellatus,* as described by Lewis et al. (2007)*.* The dissolved oxygen level was monitored using WITROX 4 and DAQ-M equipment (Loligo System, Viborg, Denmark), combined with the commercial software AutoResp (Loligo System, Viborg, Denmark). 

### Blood and tissue sampling

Right after the experiment, blood samples were quickly collected from the caudal vein, with heparinized syringes, and centrifuged for plasma separation. Then, the animals were euthanized by concussion followed by severing of the spinal cord. Gills and liver were removed and promptly frozen in liquid nitrogen. All the biological samples were stored at -80 °C for further analysis.

### Biochemical assays

Plasma glucose determination was performed with the colorimetric enzymatic glucose kit (InVitro, MG, Brazil), according to manufacturer’s instructions. For plasma lactate quantification, total plasma was acidified with 8% perchloric acid and centrifuged at 604 g for 10 min. The supernatant was removed and neutralized with 6M potassium hydroxide, and centrifuged at 604 g for 3 min. The supernatant was transferred into a microplate with glycine buffer (G5418, Sigma-Aldrich, CA, USA), β-nicotinamide adenine dinucleotide hydrate (N6522, Sigma-Aldrich, CA, USA) and L-lactic dehydrogenase (L2500, Sigma-Aldrich, CA, USA). The microplate was incubated at 37 °C for approximately 10 min. The reading was performed using a microplate reader (SpectraMax M, Molecular Devices, CA, USA) at the wavelength of 340 nm.

Liver glycogen quantification was performed according to Bidinotto et al. (1997). Liver sample (approximately 0.03 g) was placed inside a microtube containing 6N potassium hydroxide. The microtube was placed in a dry bath at 98 °C until total liver dissolution. Subsequently, 96% ethanol and 10% potassium sulfate were added to the microtube. The solution was centrifuged and the supernatant discarded. Pure water was added and used to resuspend the white sediment (Bidinotto *et al*., 1997). An aliquot was withdrawn, and 3% of phenol and sulfuric acid were added to it. The solution was mixed, and incubated for 10 min at 25 °C (Dubois et al., 1956). The reading was performed using a microplate reader (SpectraMax M, Molecular Devices, CA, USA) at the wavelength of 480 nm.

### Total RNA extraction and first-strand cDNA synthesis

Total RNA was isolated from gills and liver using TRIzol Reagent (Invitrogen, CA, USA) according to the manufacturer’s instructions. Total RNA was quantified with NanoDrop 2000 spectrophotometer (Thermo Scientific, MA, USA). The integrity of the RNA was verified by 1% agarose gel electrophoresis, showing intact 28S and 18S rRNA bands. Total RNA was diluted to a final concentration of 500 ng with nuclease-free water, and was treated with DNase I Amplification Grade (Invitrogen, CA, USA). cDNA synthesis was obtained using total RNA and High Capacity cDNA Reverse Transcription Kit (Applied Biosystems, CA, USA), according to manufacturer’s protocol. 

### Sequencing and primer obtention

A BlastN search was performed (www.ncbi.nlm.nih.gov/BLAST/) on the complete, non-redundant GenBank nucleotide database for ortholog of *phd2* in other fish species. A multiple sequence nucleotide alignment was carried out on coding sequences to design the primers. The specific sequences were obtained through the Sanger protocol using ABI 3130 (Applied Biosystems, CA, USA) following the ABI PRISM^®^ Big Dye™ Terminator Cycle Sequencing Ready Reaction (Applied Biosystems, CA, USA) protocol. The obtained sequences were analyzed through ABI 3130 Sequence Analyzer software (Applied Biosystems, CA, USA) for their electropherograms quality parameters. The contigs were generated using the BlastN validated tool for the detection of nucleotide homology on NCBI (www.ncbi.nlm.nih). Primers were designed using Oligo Explorer 1.5 software. The annealing temperature was optimized by gradient PCR. *phd2* primers sequence used in Quantitative real-time PCR (qPCR) assays was: Forward primer: 5´-AAGTTGTCGGTTAGTAGGGC-3´ and Reverse primer: 5´-TCGNTCTGCGGCTTCTCCA-3´.

### Real-time quantitative PCR

In triplicate, cDNA was used for qPCR analysis, which was performed using Fast SYBR^®^ Green Master Mix (Applied Biosystems, CA, USA) on ViiA 7 PCR-System (Applied Biosystem, CA, USA). Besides *phd2,* the primer sequences for *hif-1α* and housekeeping genes, *18S* and β*-actin*, as described by Baptista et al. (2016), were:

*18s* forward: 5´-GGGAGGTTCGAGACGATCAG-3´

*18s* reverse: 5´-TCGCTAGTTGGCATCGTTTATG-3´

β -*actin* forward: 5´-CCTTGATGTCACGCACGATT-3´

β -*actin* reverse: 5´-CAGAGCGTGGCTATTCCTTCA-3´

*hif-1α* forward: 5´-GGAGAGCACCAACGGACAA-3´

*hif-1α* reverse: 5´-GGGTCACAGATCAAAACCAGGTA-3´

The amplification of housekeeping genes was constant during the experiment, qualifying them as strong housekeeping genes for hypoxia. The efficiencies of these four genes were approximately 100%, which ensures the correct use of ΔCp equation. qPCR mix contained 5 μL Fast SYBR^®^ Green Master Mix (Applied Biosystems, CA, USA), 2 μL nuclease-free water, 1 μL cDNA, 1 μL forward primer (2.5 pmol) and 1 μL reverse primer (2.5 pmol). qPCR assays were performed under the following conditions: denaturation during 20 s at 95 °C, then 40 cycles: 1 s at 95 °C, 20 s at 60 °C, 15 s at 95 °C, with data collection after each cycle. Next, the melting curve analysis was performed to ensure the presence of a single product-specific melting temperature. To calculate the target gene relative quantification, RT-qPCR efficiency corrected calculation model was used according to Pfaffl (2004). 

### Statistical analysis

One-way ANOVA was performed to analyze physicochemical parameters of water. Relative gene expression and biochemical assays were examined for each exposure time between normoxia, hypoxia and recovery. For this, bidirectional analysis of variance (two-way ANOVA), followed by Holm-Sidak *post hoc* test, was used. Significance level was determined as α=0.05. Data were expressed as mean ± s.e.m. (standard error of the mean). Statistical analysis were performed using SigmaStat (v. 3.5) and graphics were built in SigmaPlot software (v. 11.0).

## Results

### Biochemical aspects

**Figure 1 ‒:**
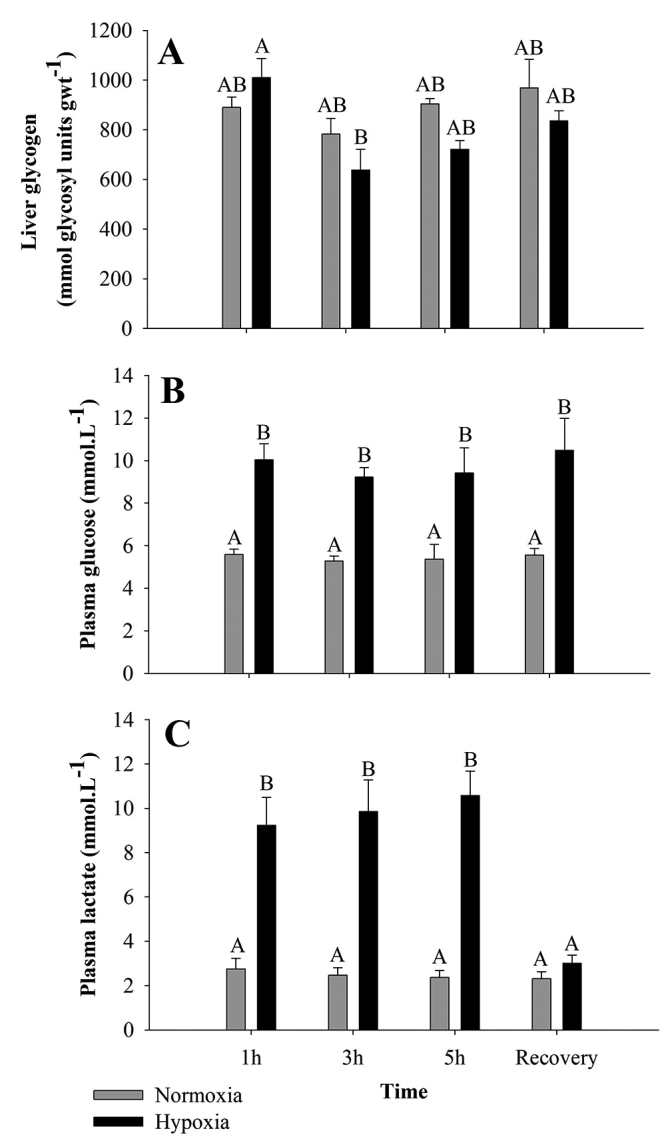
Liver glycogen (**A**), plasma glucose (**B**), and plasma lactate (**C**) levels in *A. ocellatus* exposed to normoxia (6 mg O_2_.L^-1^ ± 0.5) and hypoxia (0.7 mg O_2_.L^-1^ ± 0.05) for 1, 3 and 5 hours, and recovery (6 mg O_2_.L^-1^ ± 0.5) for 3 hours (mean±s.e.m., n=6). Statistical significance was analyzed using a two-way ANOVA. Bars with different letters indicate difference between time, normoxia, hypoxia and recovery (p< 0.05).

Exposure to 3 hours of hypoxia induced a decrease in liver glycogen when compared to 1 hour of hypoxia (p< 0.004; F = 5.12) (Figure 1A). Otherwise, after 1, 3, and 5 hours of hypoxia exposure, plasma glucose (p< 0.001; F = 59.57) (Figure 1B) and plasma lactate (p< 0.001; F = 94.12) (Figure 1C) increased, when compared to normoxia. After 3 hours of recovery in normoxia, glucose level remained elevated, but lactate returned to the original levels found in normoxia.

### Relative gene expression

**Figure 2 ‒:**
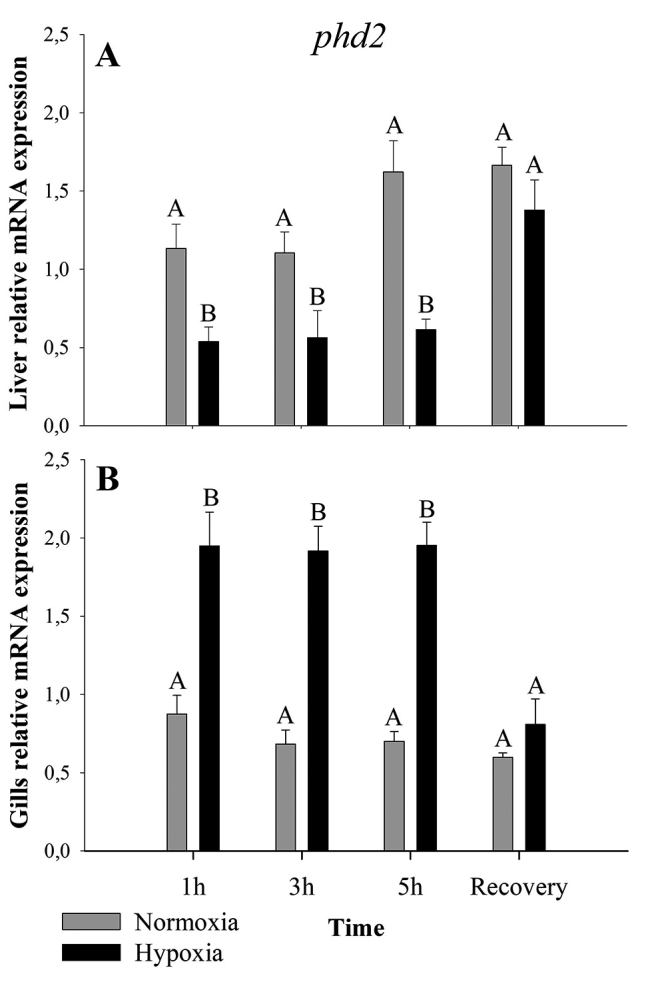
Relative expression of *phd2* in liver (**A**) and gills (**B**) of *A. ocellatus* exposed to normoxia (6 mg O_2_.L^-1^ ± 0.5) and hypoxia (0.7 mg O_2_.L^-1^ ± 0.05) for 1, 3 and 5 hours and recovery (6 mg O_2_.L^-1^ ± 0.5) for 3 hours (mean±s.e.m., n=6). Statistical significance was analyzed using a two-way ANOVA. Bars with different letters indicate difference between time, normoxia, hypoxia and recovery (p< 0.05).

Liver and gills presented inverse relative expression patterns for *phd2* mRNA levels. In liver, hypoxia caused a reduction in *phd2* transcript levels (p< 0.001; F = 33.79) compared to normoxia (Figure 2A). In gills, hypoxia increased *phd2* transcripts (p< 0.001; F = 96.28) when compared to normoxia (Figure 2B). In both cases, the recovery returned the *phd2* transcripts to normoxia levels. 

**Figure 3 ‒:**
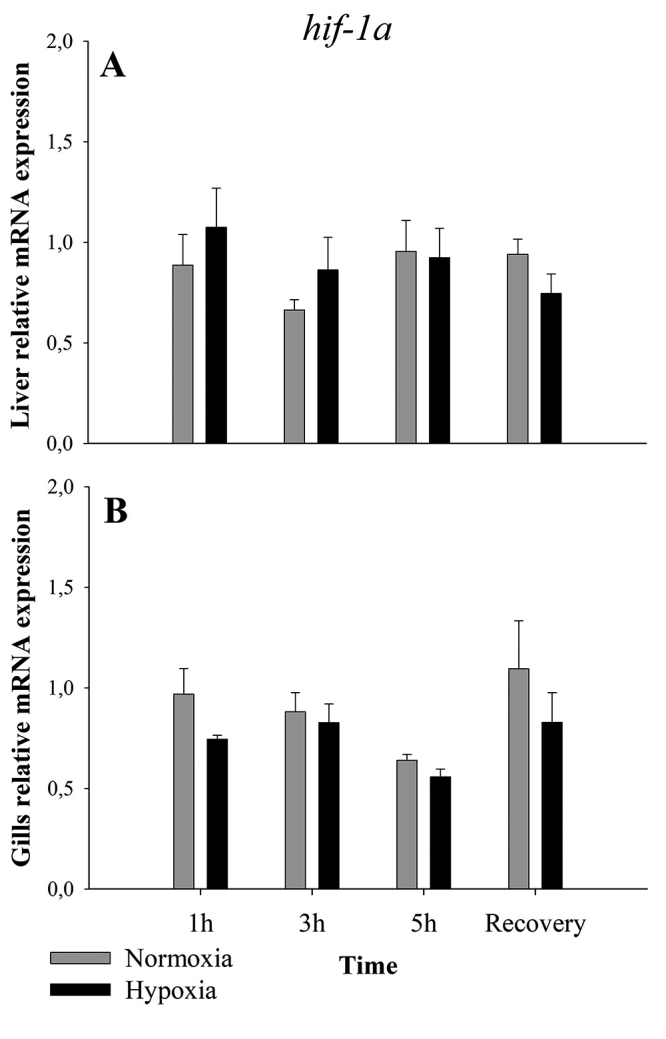
Relative expression of *hif-1α* in liver (**A**) and gills (**B**) of *A. ocellatus* exposed to normoxia (6 mg O_2_.L^-1^ ± 0.5) and hypoxia (0.7 mg O_2_.L^-1^ ± 0.05) for 1, 3 and 5 hours and recovery (6 mg O_2_.L^-1^ ± 0.5) for 3 hours (mean±s.e.m., n=5).

In liver, *hif-1α* showed no difference in expression between normoxia and hypoxia (p=0.683; F = 0.170) (Figure 3A); the same occurred in gills (p=0.101; F = 2.91) (Figure 3B).

## Discussion

With three hour of hypoxia, glycogen content decreased in liver of *A. ocellatus* (Figure 1A). Similar response has also been observed in the congeneric cichlid *Astronotus crassipinnis* by Chippari-Gomes et al. (2005). The reduction of hepatic glycogen during hypoxia is a well-known fish response to improve its tolerance to low oxygen availability (Heath and Pritchard, 1962; Chen et al., 2007; Padmavathy and Ramanathan, 2010; Chen *et al*., 2017). The hepatic glycogenolysis caused by hypoxia increases the levels of plasma glucose in order to supply energy to the body in anaerobic conditions through anaerobic glycolysis (Scarabello et al., 1992; Schulte et al., 1992; Chippari-Gomes *et al*., 2005). The same was observed with our present results, after 3 hours of hypoxia, there is a decrease in liver glycogen and an increase in plasma glucose and lactate. An increase on plasma glucose and plasma lactate levels were also observed in *A. ocellatus* under different hypoxia conditions by Muusze et al. (1998), Richards et al. (2007), Wood et al. (2007), Baptista et al. (2016) and by Heinrichs-Caldas et al. (2019) in the congeneric *A. crassipinis*. These authors observed that plasma lactate concentration, under hypoxia stress, increased, and during the recovery phase, plasma lactate returned to values close to the control. Our results indicate a glycogen mobilization, caused by hypoxic stress, along with an increase in glucose transport. The increase observed in blood lactates levels also indicate the switch to an anaerobic metabolism. 

Fishes exposed to hypoxia during 1, 3, and 5 hours, showed a *phd2* liver transcripts decrease, but increased its levels during the recovery phase, returning to similar levels found in normoxia (Figure 2A). This result contrasts with the literature, which reported an increase in liver *phd2* transcripts of other fish species submitted to hypoxia (Wang et al., 2015; Zhang et al., 2017). The lower level of liver *phd2* transcripts and, consequently, and presumably the low PHD2 protein activity, due to the hypoxia condition, allow HIF-1 protein to bind to its target genes (Rytkönen et al., 2011). Thereby, HIF-1 induces the expression of a wide variety of genes required for an organism to survive in situations where there is a lack of oxygen, as described by Wenger et al. (2005). 

As stated before, HIF-1 has several targets related to hypoxia exposition. For example, lactate dehydrogenase-A gene (*ldh-a*), one of the target genes of HIF-1 protein (Semenza, 1998, 2002; Cui et al., 2017). Thus, the reduction of *phd2* transcripts may be related to an increase in *ldh-a* transcription, as reported by Suhara et al. (2015) in mouse liver cells. Heinrichs-Caldas et al. (2019) measured LDH activity in the liver of *A. crassipinnis* submitted to hypoxia, followed by recovery in normoxia, and observed an increase in LDH activity in animals submitted to 5 hours of hypoxia and a decrease in the enzyme activity after recovery in normoxia. All these data suggest that LDH responds to hypoxia accumulating lactate, which after recovery, is converted to glycogen, as suggested by Hochachka and Somero (1984), to avoid the toxicity caused by lactate accumulation in the organs (Almeida-Val and Val, 1993). Another HIF-1 target is the *vegf*. Baptista et al. (2016) showed that *A. ocellatus* presents an increase in *vegf* transcripts levels when exposed to 3 hours of hypoxia, accompanied by an increase in *hif-1α* transcript levels, which indicates an increase in HIF-1 activity.

Gill *phd2* transcript levels increased in hypoxia exposure and returned to normoxia level during the recovery phase (Figure 2B). The increase of *phd2* transcripts in the gills during hypoxia contrasts with the data obtained by Wang et al. (2015), which verified a decrease of *phd2* expression in the gills of *Megalobrama amblycephala* submitted to hypoxia during four hours. Hypoxia allows HIF-1 protein to stabilize and to act as a transcription factor that binds to the HRE region of its target genes, such as *phd2*, that also has a HRE region (D’Angelo et al., 2003; Aprelikova et al., 2004; Metzen et al., 2005; Stiehl et al., 2006; Rytkonen et al., 2012). Consequently, we hypothesize that these high levels of *phd2* transcripts in the gills herein exposed to hypoxia were induced by HIF-1. This feedback mechanism involving *phd2* and HIF-1 protein was already observed in human cells (Marxsen *et al*., 2004; Pescador et al., 2005) and fish liver cells (Zhang et al., 2017) the same as other feedback loop found related to *phd* and *hif-1α* (Kaelin, 2005). The increased levels of the *phd2* works as an anticipation mechanism to interrupt the hypoxic responses through the degradation of HIF-1α subunit when oxygen returns to its normal level (D’Angelo *et al*., 2003; Marxsen *et al*., 2004). We believe that this feedback mechanism is related to protein synthesis, once PHD2 also hydroxylates proteins related to protein synthesis in normoxia, as the eukaryotic elongation factor 2 kinase (eEF2K). In hypoxia, due to decreased efficiency of PHD2, eEF2K will not be hydroxylated and will become capable of phosphorylating the eukaryotic elongation factor 2 (eEF2), causing a reduction of protein synthesis for ATP and amino acids preservation (Romero-Ruiz et al., 2012; Moore et al., 2015). A reduction of 50-55% in protein synthesis rates was observed in the gills of *A. ocellatus* exposed to hypoxia by Cassidy et al. (2018). Thus, we believe that the high levels of gills *phd2* transcripts during hypoxia may be important for the resumption of protein synthesis during reoxygenation. 

No differences of *hif-1α* transcripts in gills subjected to hypoxia were observed (Figure 3B). This result resembles data obtained for gills by Mu et al. (2015) and Li et al. (2017) and from other tissues of fish species (Shen et al., 2010). However, Rissanen et al. (2006), combining hypoxia with exposure to different temperatures, observed an increase in *hif-1α* transcripts in the gills of *Carassius carassius.* In our study, no changes in liver *hif-1α* transcripts was observed (Figure 3A). Mu *et al*. (2015) and Li *et al*. (2017) also did not find differences in liver *hif-1α* transcripts of other fish species exposed to hypoxia. Rissanen *et al*. (2006) also observed no differences in the levels of *hif-1α* transcripts in the liver of *Carassius carassius* exposed to long periods of hypoxia at different temperatures (18º C and 26º C). Although, Baptista et al. (2016) studying individuals of wild *A. ocellatus* with similar weight, noticed an increase in liver *hif-1α* transcripts after 3 hours of hypoxia exposure. Rytkonen et al. (2012) suggests that fish previously exposed to hypoxia amplify their transcriptional responses when compared to animals that experienced a single hypoxic stress. The hypoxia responses of *hif-1α* transcripts in our study may not reflect its protein levels, as seen by Robertson et al. (2014), which observed an increase of HIF-1α proteins even without a change in the amount of *hif-1α* transcripts. For liver, we can hypothesize that a decrease in *phd2* transcripts levels, and the reduction of oxygen concentration, is enough to stabilize HIF-1 levels, since the degradation of HIF-1α decreases, so the animal will no need to increase *hif-1α* transcript levels. 

In conclusion, hypoxia caused a decrease in liver glycogen, which was mobilized as plasma glucose to supply the switch to anaerobic metabolism, evidenced by the high levels of plasma lactate. Even though there is no change in *hif-1α* transcripts levels, the different transcription patterns of *phd2* found in liver and gills indicate that these organs have different molecular strategies to cope with hypoxia. In our study, *phd2* response to hypoxia condition was observed at the first hour and the transcripts levels were constant during the 5 hours of hypoxia in both tissues. The decrease in liver *phd2* transcript levels, combined with the lactate increase, indicate a switch from aerobic to anaerobic metabolism, since the degradation of HIF-1α should decrease. On the other hand, the increase in gills *phd2* levels can indicate two responses: (1) a prioritization of aerobic metabolism for this tissue, or (2) a preparation for reoxygenation after hypoxia, since this tissue has direct contact to dissolved oxygen. Along with these results we can say that this high hypoxia tolerant fish, *A. ocellatus*, possess a rapid and well-established mechanism to deal with low levels of environmental oxygen. 
